# Effects of Aerobic Exercise and High-Intensity Interval Training on the Mental Health of Adolescents Living in Poverty: Protocol for a Randomized Controlled Trial

**DOI:** 10.2196/34915

**Published:** 2022-01-17

**Authors:** Kean Poon

**Affiliations:** 1 Department of Special Education and Counselling The Education University of Hong Kong Tai Po, New Territories Hong Kong

**Keywords:** adolescents, mental health, exercise, socioeconomic status, intervention

## Abstract

**Background:**

The increasing rate of mental health issues among adolescents has recently been a considerable concern in Hong Kong. In particular, adolescents with low socioeconomic status (SES) are likely to experience poor mental health, including low self-esteem and high levels of anxiety, anger, and depression. Previous research has found that physical activities have a positive impact on improving mental health outcomes among adolescents. However, approximately 96% of adolescents in Hong Kong do not engage in regular exercise, which potentially increases the risk of poor mental health.

**Objective:**

In this study, we aim to examine whether changes in the 3 indicators (reduced ill-being, enhanced well-being, and cognitive functions) of mental health among adolescents with low SES are evident before and after exercise. In addition, this study compares the effectiveness of aerobic exercise and high-intensity interval training on these indicators among adolescents with low SES.

**Methods:**

A total of 78 participants from low-income families aged between 12 and 15 years from 3 to 4 secondary schools will be recruited for this study. They will be randomly assigned to either an aerobic exercise group (26/78, 33%), a high-intensity interval training group (26/78, 33%), or a control group (26/78, 33%). Participants in the first 2 groups will take part in a 10-week training program period. Participants in the control group will participate in other physical activities during the same intervention period. The training sessions will be conducted 3 times per week on nonconsecutive days. A range of neuropsychological tests and psychometric scales will be used to measure the executive functions and indicators of psychological well-being and ill-being, including enjoyment, self-efficacy, mood, depression, anxiety, and stress at pretest, posttest, and follow-up assessments.

**Results:**

The project was funded in 2021 by the Research Matching Grant Scheme, through the University Grants Committee of the Hong Kong Special Administrative Region Government. Ethical approval has been obtained from the author’s institution. Participant recruitment will begin in January 2022 and continue through to April 2022. Data collection and follow-up are expected to be completed by the end of 2022. The results are expected to be submitted for publication in 2023.

**Conclusions:**

The findings will help inform policy makers and practitioners in promoting the importance of physical exercise to enhance mental health.

**Trial Registration:**

ClinicalTrials.gov NCT050293888; https://clinicaltrials.gov/ct2/show/record/NCT05029388

**International Registered Report Identifier (IRRID):**

PRR1-10.2196/34915

## Introduction

### Mental Health in Adolescents in Hong Kong

Poor mental health among adolescents has serious implications for adult morbidity and mortality. Mental health involves the absence of mental illnesses and life satisfaction [1]. In Hong Kong, the mental health of adolescents has been a subject of considerable concern in recent years. A recent survey of 11,493 local citizens revealed that approximately 74% of adolescents aged <25 years exhibit moderate to high levels of depressive symptoms (eg, depression, anxiety, and stress), 41% of the adolescents exhibit moderate to high levels of posttraumatic stress symptoms, and 36% of the adolescents have both diagnoses [2]. However, previous research has demonstrated that poor mental health is unevenly distributed across different socioeconomic groups, with high clusters in families with a low socioeconomic status (SES) [3].

Families in this category are deprived in multiple ways and are affected by numerous stressors related to finances, social relations, employment opportunities, and physical illness compared with families from other socioeconomic groups [4]. For instance, adolescents with low SES often have worse access to education and social participation than their peers with an average or high SES [5]. In addition, the results from a meta-analysis study of 34 countries from 2002 to 2010 indicated that adolescents with low SES are affected by physical symptoms and have poor mental health, including low self-esteem and high levels of anxiety, anger, and depression [6]. In a sample of Chinese adolescents in Hong Kong, adolescents who grew up in families with a Comprehensive Social Security Assistance (CSSA) scheme had significantly higher levels of suicidal intention than those in families without CSSA [7].

### Interventions for Mental Health

Numerous mental health interventions are popular, but the scope of their effects is unclear. Costigan et al [8] stated that effective mental health interventions should address the following 3 indicators: (1) cognitive function, (2) well-being, and (3) ill-being. Conventional strategies such as cognitive behavioral therapy (CBT) [9] and dialectical behavior therapy (DBT) [10] are mainly based on therapeutic models. CBT focuses on challenging and changing dysfunctional thoughts, and DBT encourages clients to accept and validate their feelings [9]. Although the results in terms of reducing clinical symptoms are promising, their effectiveness in enhancing overall well-being is debatable. Moreover, one has to commit themselves to the process to benefit from CBT or DBT; these strategies may not be suitable for individuals with low capacity to change themselves (their thoughts, feelings, and behaviors). Moreover, school-based prevention programs [11] and web-based self-help interventions [12] are commonly used as preventive measures in school settings. School-based programs are implemented in schools to minimize high-risk behaviors [11]. Web-based self-help interventions allow individuals to work in their own time and use web-based resources to assist with their problems [12]. Similar to conventional strategies, the effectiveness of these intervention programs relies heavily on an individual’s motivation and capacity. Individuals with low SES often perceive themselves as incompetent, and this stigma contributes to their low motivation to change [13]. Furthermore, several studies have criticized that prevention programs fail to target the enhancement of mental health among *at-risk* students and to train school personnel, such as teachers and social workers, to identify these students [14]. Therefore, physical exercise is presently considered as an effective intervention because of its safe, nonpharmacological, and cost-effective nature [15] that can provide a range of health benefits, including improvements in body composition and physical capacity, among individuals [16]. Recent evidence has confirmed that physical exercise has a positive effect on mental health outcomes for youth through physiological and psychological pathways [17].

### Previous Research on Physical Exercise

#### Physiological Evidence

Studies that are focused on physiological evidence have commonly demonstrated a direct positive effect of physical exercise on individuals’ neurological processes, thereby indicating that cardio activities are directly and immediately beneficial to mental health. Two main research streams that cite physiological evidence, namely, those that focus on monoamines and those that focus on endorphins, are present. The first research stream reveals that physical activity upregulates the synaptic transmission of monoamines, including the 3 major neurotransmitters, namely, norepinephrine, dopamine, and serotonin [18]. A similar effect has been found for antidepressant drugs [19], although exercise has been proven to be as effective as antidepressants for alleviating depressive symptoms among patients with major depression [20]. The hypothesis regarding endorphins is also popular for explaining the impact of aerobic exercise on mental health. Several researchers have believed that endorphins may lead to energy conservation during exercise and consequently exhibit psychological effects, such as improved mood states and reduced anxiety [21]. Direct evidence stating that physical activity can elevate plasma endorphin levels also exists [22].

#### Psychological Evidence: 3 Mental Health Indicators

Although physiological evidence has established a direct association between physical activity and mental health, researchers are also keen to identify how physical activities may benefit mental health. Several researchers have conducted studies based on the three indicators of mental health that were proposed by Costigan et al [8]: (1) cognitive function, (2) well-being (eg, enjoyment), and (3) ill-being (eg, depression and negative affect).

With regard to cognitive function, growing evidence indicates that participating in exercise positively affects the executive functions [23]. Executive function is an umbrella term that covers a wide array of cognitive processes that govern goal-directed actions and adaptive responses to novel, complex, or ambiguous situations [24]. Numerous studies have found that intense physical exercise improves working memory [25], inhibitory control [25], and cognitive flexibility in typical [26] and low-income adolescents [27].

Physical exercise has been associated with improved well-being, including life satisfaction and self-esteem [8]. Exercise is a challenging activity, but the process of engaging in exercise could provide individuals with a meaningful experience of mastery that may lead to improved mood states and self-confidence [28]. Affect regulation theory suggests that exercise could regulate affect by reducing negative mood states and enhancing positive mood states that are conducive to mental well-being [29-31]. Furthermore, as described in the relevant literature, exercise can divert an individual from unfavorable stimuli (eg, worries, stress, and depressive thoughts), leading to improved mood [32,33].

Physical exercise has also been associated with reduced ill-being. A meta-analysis of 73 studies confirmed that increased levels of physical activity significantly reduced depression, anxiety, psychological distress, and emotional disturbance among children [34]. Archer and Garcia [35] found that regular physical exercise ameliorated the symptoms of anxiety and depression. Behavioral activation theory posits that depressive symptoms may be alleviated when individuals replace passive activities with exercise and other entertaining activities [36].

### Exercise Regimen

Although a lack of consensus regarding the most effective training regimen for physical exercise is evident in clinical review studies from 2000 to 2014, Ranjbar et al [37] have recommended the following components of an effective exercise program to benefit adolescents with poor mental health. On the basis of the frequency, intensity, time, and type principle, an exercise program should include the following characteristics: (1) structured aerobic exercise, which requires the heart to pump oxygenated blood for delivering oxygen to working muscles and stimulates an increase in the heart rate (HR) and breathing rate [38]; (2) group exercise, particularly for adolescents; (3) low (40%-55% maximal oxygen consumption) to moderate (65%-75% maximal oxygen consumption) intensity exercise; (4) 45- to 60-minute exercise sessions; (5) exercise frequency of at least three to four sessions per week, which is equal to ≥150 minutes of exercise per week; and (6) an exercise duration equal to ≥10 weeks.

Overall, extensive research has confirmed that physical exercise can significantly improve physical and mental health, whereas aerobic exercise has been the main focus of most previous studies. Recent studies have attempted to determine whether low-volume high-intensity interval training (HIIT) could be a time-efficient exercise strategy for improving health and fitness among the general population.

### High-Intensity Interval Training

HIIT is a time-efficient type of aerobic training that involves a short duration of full-effort exercise, followed by a rest period. HIIT is mainly appealing because it can be completed in a relatively short period and results in physiological adaptations that are equivalent to long sessions of traditional aerobic training and improvements in physical and mental health. This strategy may be feasible and effective for increasing physical health outcomes among young people [39,40]. Compared with low-intensity, high-volume (duration) endurance aerobic training (ie, cycling), HIIT can result in better oxygen uptake, greater muscle deoxygenation, and better exercise performance [41]. A previous study also revealed that patients participating in high-intensity interval running reported higher perceived enjoyment than those participating in moderate-intensity continuous running [42]. A pilot study of a 10-week HIIT program resulted in improved metabolic outcomes among patients with schizophrenia, thereby supporting the benefits of HIIT for physical fitness and mental health. Furthermore, studies have also shown that HIIT reduces distress and anxiety [43].

Despite the promising evidence that supports the adoption of HIIT among adults with various conditions, limited research that targets adolescents, particularly those with low SES, is available [39,40]. High-intensity activities performed in short, repeated bouts with periods of recovery in-between could be an achievable and enjoyable alternative to high-volume continuous exercise for adolescents, including *low-active* adolescents [40,44].

### Research Gaps and Study Objectives

The conditions and ability of poor individuals to manage and potentially escape poverty are the core of policy agendas and ethical and economic concerns among societies. Behavioral research has recently been applied to policy-relevant challenges and has explored the significant potential of simple interventions to influence cognition and behavior [45]. Poor individuals, particularly young poor individuals in Hong Kong, have received little attention as part of this endeavor despite their characteristics that satisfy the qualifications of clear candidates for interventions that could improve disadvantaged situations.

Although several longitudinal studies have confirmed that growing up in a disadvantaged family is a significant risk factor for poor mental health, numerous research gaps should be addressed by further investigation. For instance, most studies on mental health and socioeconomically disadvantaged adolescents have been restricted to understanding and confirming the link between mental health and SES. Research that aims to examine whether mental health can be enhanced through physical exercise is scarce. However, such research would be extremely useful for expanding early interventions. To date, a steady decline in the number of physically active students in Hong Kong is evident. Furthermore, the situation among adolescents in Hong Kong is worse, given that 96% of them are insufficiently active [46]. Hence, there is a considerable need for this kind of research to arouse public awareness of the link between physical activity and mental health. Although previous studies have reported improvements in the mental health of adolescents after exercise sessions, a lack of follow-up data is available on assessing the sustainability of exercise intervention with moderate intensity over a long duration. In addition, relatively few studies have directly compared the effects of aerobic exercise and HIIT on the mental health of adolescents with low SES. Given that HIIT training has become popular and effective in improving health and well-being, HIIT exercise should be used as the comparison group to examine the effectiveness of traditional aerobic exercise.

Following the health benefits of regular exercise and the limited research on exercise-based interventions (aerobic exercise vs HIIT) in low-SES adolescents, this study primarily aims to investigate the effectiveness of aerobic exercise and HIIT on the 3 indicators of mental health among low-SES adolescents. This study particularly intends to answer the following two research questions: (1) Is there any change in the 3 indicators of mental health (ie, cognitive function, well-being, and ill-being) before and after exercise? and (2) Which exercise regimen (aerobic exercise or HIIT) is more effective in reducing ill-being and enhancing well-being and cognitive function among adolescents with low SES?

## Methods

### Overview

Participants will be randomly assigned to an aerobic exercise group, an HIIT group, or a control group. Participants in the first 2 groups will partake in a 10-week training program, conducted 3 times per week on nonconsecutive days. Participants in the control group will participate in other physical activities during the same intervention period.

### Study Design

A randomized controlled trial will be used to examine the effects in individuals who are assigned to either a 10-week aerobic exercise training group, a 10-week HIIT training group, or a control group. A total of 78 secondary school adolescents will be recruited for this study. Subsequently, the adolescents will be randomly clustered in a controlled intervention trial. The effectiveness of the intervention will be assessed through a pretest, posttest, and a follow-up design that will be evaluated based on the degree of improvement in performance across the 3 time points. If any important protocol modifications are made, all relevant parties will be informed. These include the trial registry, human research ethics committee, research team members, all participating students and their parents, the journal that publishes the study protocol, and the funding body.

### Participants

G*Power [47] was used to estimate the sample size based on analysis of variance (ANOVA) with Cohen *f* [48] medium effect size set as 0.25 and Cronbach α, as .05. A total of 45 participants are required to achieve sufficient statistical power. Assuming a 20% attrition rate [49] and clustering effect (assumed as 0.01) [50], the principal investigator (PI) will gather a total of 78 participants aged between 12 and 15 years from 3 to 4 secondary schools in Hong Kong. Each group will comprise 33% (26/78) of participants.

We will conduct the study across 3 to 4 secondary schools that will be randomly selected. Students will be invited to participate in the study given that they (1) are aged between 12 and 15 years and (2) belong to a family with a household income below half of the median household income reported in Hong Kong, which is adjusted by household size. These selection criteria are based on a study by Costigan et al [8], which examined the effect of HIIT on cognitive and mental health among adolescents. Participants (1) with hypertension or diabetes mellitus without control; (2) with a history of brain injury and other neurological diseases, epilepsy, or myocardial infarction; (3) with recent musculoskeletal disease; (4) who use medication that affects HR (eg, *β*-blockers, asthma medications, stimulants, digoxin, and antiarrhythmic agents); or (5) with cardiovascular disease will be excluded from this study. All participants in the exercise groups must complete a Chinese version of the Physical Activity Readiness Questionnaire for a safe preliminary screening before the exercise class [51].

### Procedures

**Figure 1:**
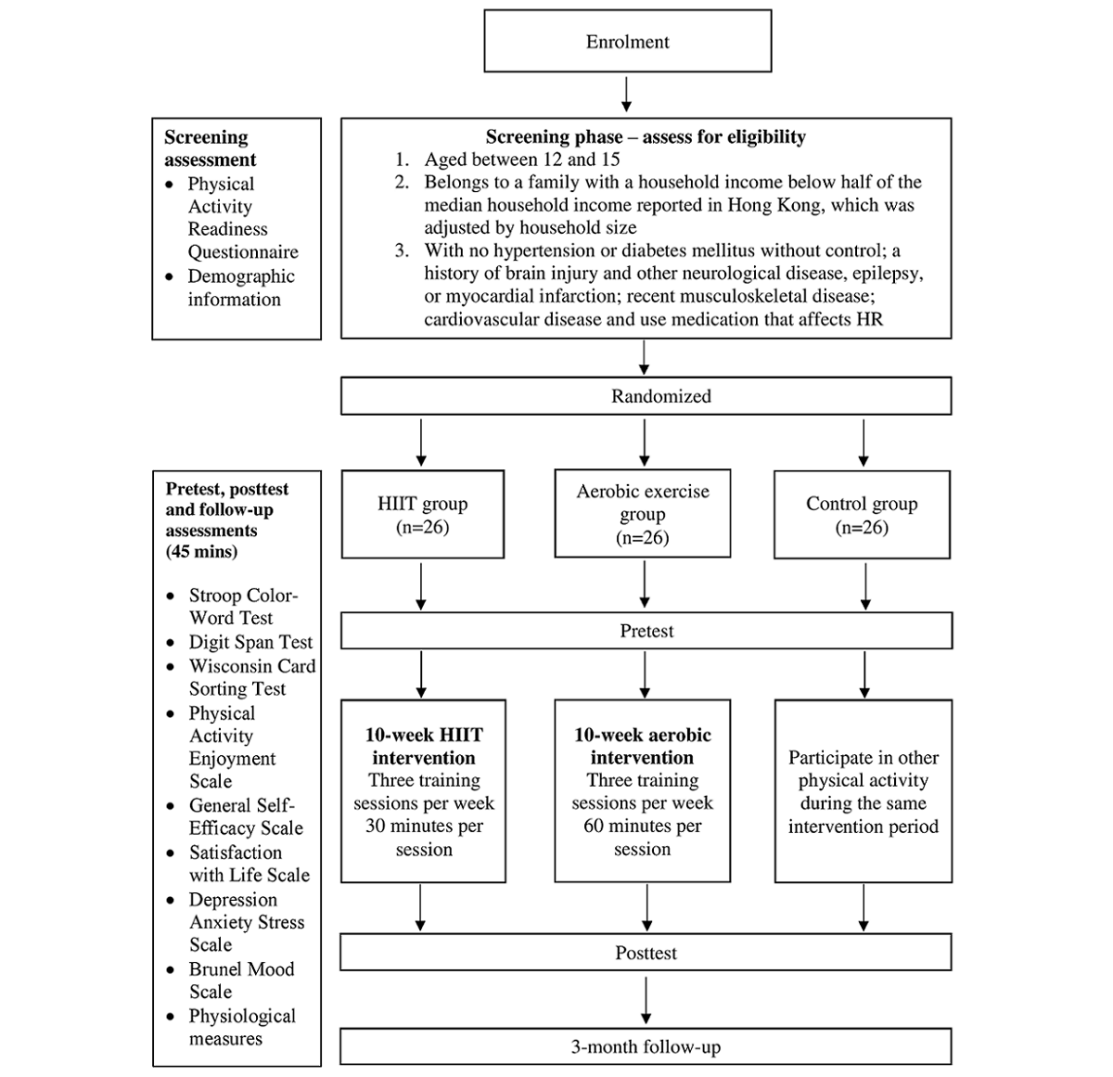
Flow diagram: study design and data collection procedures. HIIT: high-intensity interval training; HR: heart rate.

After obtaining ethical approval from the institution, the author will contact the principals of the selected secondary schools to seek their permission to recruit participants from the pool of students. A research assistant will obtain informed consent forms and the Physical Activity Readiness Questionnaires from the participating students and their parents. Before the intervention, the students will be asked to complete the baseline assessment for the exercise programs. They will be asked to perform the assessment again after completing the exercise programs. A cluster design will be used for all the participants in the same school to delegate them to the same combination of 2 intervention groups and a control group. The participants will be randomly assigned to 1 of the 3 groups. The trial is designed to simultaneously evaluate the impact of the 2 interventions. Randomization will be performed using computer-generated random numbers by a trained research assistant blinded to the identities of the participants. The research assistant conducting the randomization will not be involved in any data collection. The entire process of data collection and intervention will be conducted by other trained research assistants who are blinded to the participants’ group assignments. A follow-up assessment will be conducted 3 months after the exercise program to examine the long-term effects of such an intervention. All assessments will last for 45 minutes. All participants will be given an exercise diary or logbook to keep track of their exercise habits (ie, record the type of exercise or activity, hours, and intensity of exercise or activity every day) throughout this study (Figure 1).

### Aerobic Exercise Group

Following the exercise recommendation in the literature review and the study by Ranjbar et al [37], this study will conduct the aerobic exercise classes over a period of 10 weeks. The participants will complete 3 training sessions per week. In addition, each aerobic training program will last for 1 hour. The session will include a 10-minute warm-up period, followed by a 45-minute aerobic workout, wherein the participants can choose to use either a treadmill, a cycle ergometer, or a rowing ergometer. After the workout, a 5-minute period of stretching will be provided for cooling down. The intensity during the workout will be set at 40%-55% of the individual’s maximum HR [37].

### HIIT Group

The HIIT protocol has been adopted from the studies by Giannaki et al [52] and Wu et al [43] and has been slightly modified. The intervention will be conducted over a period of 10 weeks, and the participants will complete 3 training sessions per week. Each HIIT training program will last for 30 minutes. The session will include a 10-minute warm-up period, followed by a 15-minute HIIT workout, and a 5-minute period of stretching to cool down. The exercise will include repeated, high-intensity intermittent bursts of vigorous activity at maximal effort. The HIIT will mainly include bodyweight exercises (eg, push-ups, squats, and lunges) that disregard equipment use. The intensity during the workout will be set to 85%-95% of the individual’s maximum HR (Textbox 1). The exercise was designed by a registered physiotherapist with more than 10 years of clinical experience. A half-day training workshop will be provided to the personal trainer to ensure that they know the exercise and can demonstrate them to the participants.

**Design of the high-intensity interval training (HIIT) program.**

**Study design for HIIT**

- Start: 10-minute warm-up.
- HIIT program: Every circuit takes 3 minutes 45 seconds.
- Work: 15 seconds.
- Rest: 10 seconds.
- Work: 15 seconds.
- Rest: 20 seconds.
- Work: 15 seconds.
- Rest: 30 seconds.
- Work: 15 seconds.
- Rest: 40 seconds.
- Work: 15 seconds.
- Rest: 50 seconds.
- Repeat 4 circuits.
- End: 5 minutes of stretching.

The *work* period indicates the short-duration, full-effort exercise to boost the heart rate (HR) to 58%-95% or to the maximum HR.

The *rest* period indicates the active recovery with light stepping or *jumping jack* exercise at the effort to achieve 40%-50% of the maximum HR [53].

The HIIT and aerobic exercise groups will be led by a certified personal trainer with at least three years of experience in guiding adolescents in group exercise. In terms of the duration of intervention for the HIIT and aerobic exercise groups, research has shown that approximately 30 minutes is the ideal session length for an HIIT work out with an HR >90% for 3 days per week over a period of 10 weeks. For aerobic exercise, research has revealed that participants who exercise 3 times per week for 45-minute sessions over 10 weeks exhibited improved mental health. By contrast, exercising for >90 minutes per session could lead to poor mental health, which includes physical and mental exhaustion [54]. To prevent symptoms of overreaching (eg, fatigue, mood disturbance, and disrupted sleep), the aforementioned intensity and duration of the exercise will be applied in this study. Aerobic exercise and HIIT training will be conducted in the fitness room of a local sports center hosted by the Leisure and Cultural Services Department in Hong Kong [55].

### HR Monitoring

To monitor the intensity of the training during the program, the participants are required to wear HR monitor watches (Polar Electro Oy). The recorded HR data will be uploaded to a computer and will be checked regularly by investigators to determine whether the participants could reach the target HR during the exercise. Furthermore, the length of time (number of minutes and average session duration) when the participant reaches the HR targets will be recorded. The participants will also be encouraged to achieve the target HR during the session. The maximum HR will be calculated according to the age-based formula (220-age). To ensure their safety and manage unexpected situations, a subjective measurement of physical exertion (using Borg scale 6-20) will also be monitored during every training session by an assistant trainer [56].

### Promoting Participants’ Adherence to Exercise

Given that adolescent girls and boys alike may have difficulty in starting and adhering to a regular exercise program, several approaches will be conducted to promote their adherence to such programs. First, to develop an enjoyable session, fun warm-up and cool-down activities or games will be included, and participants will work with their preferred partner. To create a supportive environment, a reward and peer support system will be incorporated (eg, for pairs who provide verbal encouragement and support their peers and for their hard work during the HIIT sessions). In addition, a certificate will be awarded to recognize the participants who exert outstanding effort and dedication during the workout. Thus, positive feedback and motivation are encouraged among the partners. To promote autonomy, participants will be allowed to choose the music, select particular exercises to be completed during the workout, and choose a workout when the exercise has been mastered [39]. Participants’ responses to the intervention and reasons for early withdrawal during the intervention will also be documented in the intervention logs and the content analyzed.

### Outcome Measures

The SES data will be obtained from 2 independent sources, namely, self-reported parent questionnaires (including parents’ income and education level) and the school-reported government subvention record. Parental education will be assessed based on a 4-point scale, ranging from 1 (*primary school*) to 4 (*master’s degree or above*). The family’s monthly income will also be measured using a 23-option item with options in Hong Kong Dollar from 1 (*none*) to 23 (HK$ 32,000-$ 33,999; US $4000-$4400).

The low-SES category will be constructed based on the calculation of the income-to-needs ratio, which will be calculated by dividing the total family income by the poverty threshold for the appropriate family size following the Hong Kong government’s calculation. Families living below the poverty line with an income-to-needs ratio between 0 and 1 [57] will be classified as having low SES. The question of whether they have received financial income from several sources, including wages, family members or friends, a CSSA, an old age allowance and other government assistance, pensions, rental income, investment income, and other income will also be asked as supplementary information.

### Measures of the 3 Indicators of Mental Health

#### Cognitive Function

A range of neuropsychological tests from the Millisecond software [58] will be administered to the participants. A computerized battery of neuropsychological tests to measure the 3 core executive functions would include the Stroop Color-Word Test (inhibition), the Digit Span Test (working memory), and the Wisconsin Card Sorting Test (cognitive flexibility). These neuropsychological tests have been proven to be valid and reliable measures of adolescents’ cognitive functions in previous research [59]. The author has been using these 3 executive functioning tests to measure adolescents’ cognitive functions [60].

#### Well-Being

##### Enjoyment

A short Chinese version of the Physical Activity Enjoyment Scale (S-PACES) [61] will be used to measure their enjoyment before and after exercise. The S-PACES includes 7 negatively worded items (eg, “It is not at all interesting”) anchored on a 5-point Likert scale that ranges from 1 (*disagree a lot*) to 5 (*agree a lot*). The internal consistency of the S-PACES is high (Cronbach α=.96) [61].

##### Self-efficacy

A Chinese adaptation of the General Self-Efficacy Scale [62] will be administered to measure the adolescents’ self-efficacy. The General Self-Efficacy Scale contains 10 items (eg, “I can constantly manage to solve difficult problems if I try hard enough”), with responses ranging from 1 (*not at all true*) to 4 (*exactly true*). The internal consistency and reliability are 0.76-0.90 and 0.80, respectively.

##### Life Satisfaction

The Chinese version of the Satisfaction with Life Scale [63] will be used to measure life satisfaction. This scale is a short 5-item (eg, “I am satisfied with my life”) scale based on a 7-point rating scale that ranges from 1 (*strongly disagree*) to 7 (*strongly agree*). The Satisfaction with Life Scale has demonstrated a good reliability coefficient of 0.82 [63] and has been used in previous research using Chinese samples (Cronbach α=.84) [64].

#### Ill-Being

##### Depression, Anxiety, and Stress

The Chinese version of the 21-item Depression Anxiety Stress Scale [65] will be used to measure depression (eg, “I felt that I lack something to look forward to”), anxiety (eg, “I felt that I was close to panic”), and stress (eg, “I found it difficult to relax”) among adolescents. The respondents will be asked to rate the items using a 4-point combined severity-frequency scale that ranges from 0 (*not applicable to me at all*) to 3 (*extremely applicable to me*) based on their experiences related to each item over the past week. The Cronbach α for the depression, anxiety, and stress subscales is .83, .80, and .82, respectively, which indicates good reliability. The 21-item Depression Anxiety Stress Scale has been used in a previous study involving a Chinese sample [66].

##### Mood

The Chinese version of the Brunel Mood Scale [67] will be created based on the study by Terry et al [68] to assess the mood among Chinese students and adults. The Chinese version of the Brunel Mood Scale is a 23-item inventory with the following 5 negative mood dimensions: anger, confusion, depression, fatigue, and tension. Respondents will be asked to indicate their feelings (eg, angry, unhappy, or nervous) using a 5-point Likert scale that ranges from 0 (*not at all*) to 4 (*extremely*). All subscales have achieved good internal consistency and reliability (Cronbach α=.74-.85).

### Physiological Outcome Measures

The physical health outcomes survey will include measures of blood pressure (BP), resting HR, body weight (BW), height, waist circumference (WC), and hip circumference (HC). BP will be measured using an automated BP monitoring device. The participants will be given a 10-minute rest before the measurement. The BP will be measured twice, with a 1-minute interval between the readings. Additional readings will be obtained if the BP measurements differ by >5 mmHg. The mean of the 3 consecutive readings will be used as the examination value. The resting HR will be measured through a wireless HR monitor that will be worn by the participants (Polar Electro Oy) for 1 minute after their 10-minute rest. BW and height will be measured using an electronic digital scale and a stadiometer, respectively. BMI will be calculated by dividing BW (kg) by height (m^2^). The WC and HC measurements will be adopted from the study by Bacopoulou et al [69]. The WC and HC will be measured twice using an anthropometric tape while the adolescents stand erect and relaxed with their arms at their sides and feet positioned close together. The WC will be measured between the upper border of the iliac crest and the lowest border of the rib cage at the end of normal expiration. The HC will be measured at the widest part of the hip at the level of the greatest trochanter. For all the measurements, the tape will be positioned at a level that is parallel to the floor. Furthermore, the unit of measure will be in centimeters to the nearest 0.1 centimeter. The waist-to-hip ratio will be calculated as the ratio of the waist-to-hip circumference. All physical data will be recorded in the resting state at the baseline each week and at the end of training. The measurement for the physical health outcome assessment will be performed by a trained research assistant.

### Data Analysis

Interim analysis will not be performed, and data analysis will only begin after the follow-up stage. The PI and trained research assistants will have access to the final trial data set. Data will be analyzed using SPSS version 26. A missing value analysis procedure will be conducted to detect the missing values in the data file. The demographic characteristics of the participants will be investigated using the chi-square test for independence (for nominal variables) and 1-way ANOVA (for continuous variables). A Bonferroni post hoc test will subsequently be conducted for group comparisons. To examine the changes between and within groups for each outcome measure (ie, self-efficacy, mood, enjoyment, working memory, cognitive flexibility, inhibition, depression, anxiety, and stress), a 3 (before intervention vs after intervention vs follow-up) × 3 (aerobic exercise vs HIIT vs control) repeated-measure ANOVA will be conducted. A post hoc analysis will be used to confirm whether significant differences for any of the outcome variables exist among the groups. The Bonferroni method will be used to adjust the *P* values.

### Declarations

All of the study procedures have been examined and approved by the Human Research Ethics Committee of the Education University of Hong Kong (reference number: A2019-2020-0167). Before the commencement of the study, oral assent will be obtained from the participating students and written informed consent, from their parents by a trained research assistant. A 6-digit number will be assigned to each participant and marked on all assessments to enable identity linkage among the pretest, posttest, and follow-up assessments. All the information obtained will be handled only by the PI and trained research assistants for research purposes. All data will be kept strictly confidential and will be identifiable by codes known only to the researchers. The signed consent forms and completed questionnaires will be stored separately. Entered data will be stored on a password-protected computer, whereas original, anonymized hard copies of the questionnaires will be stored in a locked office at the Education University of Hong Kong for 5 years after publication.

## Results

This study was funded in 2021 by the Research Matching Grant Scheme through the University Grants Committee of the Hong Kong Special Administrative Region Government. Ethical approval has been sought from the Human Research Ethics Committee of the Education University of Hong Kong. Study enrollment has been delayed owing to the COVID-19 pandemic. Participant recruitment will begin in January 2022 and continue through to April 2022. Data collection and follow-up are expected to be completed by the end of 2022. The results are expected to be submitted for publication in 2023. Knowledge exchange seminars and workshops will be held at the participating schools and the author’s institution. The research findings will be presented at local and international conferences for large-scale public dissemination. The results of this study will be published in significant journals. The full protocol, participant-level data set, and statistical code will be made publicly available after the completion of the study.

## Discussion

This project aims to examine whether aerobic exercise and HIIT can improve cognitive functions and well-being (eg, enjoyment) and reduce ill-being (eg, negative affect) among adolescents with low SES. Measures of subjective well-being indicators can be beneficial in examining the need for certain policies and measuring the outcomes of policy interventions. The positive findings of this study may yield a low-cost and effective intervention that enhances the overall mental health of adolescents, particularly of those living in low-income families. Despite these strengths, there are some limitations that must be considered in this study. It is anticipated that only participants with a high level of interest and motivation will enroll in exercise training. The study could be discontinued because of the current COVID-19 pandemic situation and some participants may drop out. To ensure adequate statistical power to detect statistical significance, a 20% dropout rate will be taken into account when estimating the initial sample size before the intervention. In summary, this study demonstrates the important role of regular exercise—during or after school—on the mental health of adolescents. Ultimately, we believe that such efforts will inspire policy makers to realize the benefits of physical activities at school and the importance of enabling students to integrate physical exercise into their lives.

## Appendix

Multimedia Appendix 1 Peer-review report by The Education University of Hong Kong-Committee on Research and Development.

## Abbreviations

ANOVA: analysis of variance
BP: blood pressure
BW: body weight
CBT: cognitive behavioral therapy
CSSA: Comprehensive Social Security Assistance
DBT: dialectical behavior therapy
HC: hip circumference
HIIT: high-intensity interval training
HR: heart rate
PI: principal investigator
SES: socioeconomic status
S-PACES: Physical Activity Enjoyment Scale
WC: waist circumference
