# Supplementary material for: Effects of Aerobic Exercise and High-Intensity Interval Training on the Mental Health of Adolescents Living in Poverty: Protocol for a Randomized Controlled Trial
Source: JMIR Res Protoc. 2022 Jan 17;11(1):e34915. doi: 10.2196/34915 (PMC8804952; doi:10.2196/34915)
Supplement: Multimedia Appendix 1 [file resprot_v11i1e34915_app1.pdf]

The Education University of Hong Kong
Committee on Research and Development

Comments from Reviewers

Project Title: Effects of Aerobic Exercise and High-Intensity Interval Training on the Mental Health of Adolescents Living in Poverty

Principal Investigator: Dr Poon, Kei Yan

Proposal Assessment

The project:

Scientific/ scholarly merit:	Good
Duration proposed:	Appropriate
Impact of Research:	Low

The principal investigator:

Ability to undertake the proposal:	Very Good
Track record in field:	Very Good

Overall Recommendation: **Good**

DETAILED COMMENTS

1. The objective(s):

If physical activity has been shown to have positive effect on adolescent's mental health, the impact of the studying for knowing the effects of aerobic exercise and high-intensity interval training on mental health of adolescents will be low.

2. Policy relevance of the proposal on public policy in Hong Kong:

If the use of high-intensity interval training (HIIT) is demonstrated to be useful for enhancing mental health in adolescents with socioeconomic status (SES), that may be good to be promoted in schools.

3. The Background of research, Research Plan and Methodology:

1. The proposal should be carefully checked as many critical references could not be found in the reference list.
2. In recruiting the participants, will it result in any labeling effect that they are from SES?
3. The effect of the exercise may be transient if the participants could not develop regular exercise habit after the study. The carryover effect of the exercise program will be more important.
4. The exercise program may not able to last for too long due to busy study schedule of HK students.
5. Was the sample size estimated based on the pilot data? The sample size estimated is 79, why does the study propose to use 108 subjects?
6. A participant with household income above half of the median household income report in HK may not be a SES.
7. Will there be any control of the exercise habit prior to joining the program?

4. The feasibility of the proposed research:

The project team may need to tackle the following technical issues:
1. If the participants are not willing to disclose their family monthly income, will it be ethical for the school to disclose the subvention record?
2. The inclusion criteria may not be able to exclude participants with hidden cardiac disorder. This may impose a risk for the application of HIIT.

5. The most original or innovative aspect of the proposed research; the research result bring about to the related field if the proposed research is successful:

The most innovative aspect of the study is the use of HIIT as a time efficient exercise program for school children.

6. The reasonableness of the proposed budget, manpower planning and project duration:

Seems reasonable.

7. Overall Assessment
a) Overall Comments:

If physical activity has been shown to have positive effect on adolescent's mental health, the impact of the studying for knowing the effects of aerobic exercise and high-intensity interval training on mental health of adolescents will be low.

b) Strengths:

The most innovative aspect of the study is the use of HIIT as a time efficient exercise program for school children.

c) Weaknesses:

3. The effect of the exercise may be transient if the participants could not develop regular exercise habit after the study. The carryover effect of the exercise program will be more important.

d) Suggested improvements:

The proposal could be improved if the carryover effect of the exercise program is evaluated.

Proposal Assessment

The project:

Scientific/ scholarly merit:	Fair
Duration proposed:	Too Short
Impact of Research:	Low

The principal investigator:

Ability to undertake the proposal:	Good
Track record in field:	Good

Overall Recommendation: **Fair**

DETAILED COMMENTS

1. The objective(s):

If the objective to show that aerobic exercise and high intensity have greater effects on 3 aspects of mental health than no exercise then a simple pre-post intervention design does not allow any long-term impact.

2. Policy relevance of the proposal on public policy in Hong Kong:

The proposal seems like an efficacy study because there is no mention of how such an intervention would be targeted (within schools?) at, or implemented with, low SES children, and what the expected reach (ie, acceptability and feasibility) would be for children who, as the proposal explains, are already less active than children from higher SES backgrounds. Without knowing how many children invited are likely to interested it is hard to estimate the potential impact should the findings some degree of efficacy.

In the proposal, I would like to see a stronger link between the evidence for existing public health interventions and the effects that could be shown from this study. For example, if children from low SES backgrounds are at greater risk of depression, anxiety and perhaps substance misuse, and current interventions have limited evidence of effectiveness (over 6-12 months or longer) then perhaps a stronger case could be made for the proposed study. With a design which just assesses pre-post intervention changes the case for investing in such an intervention would be quite limited.

3. The Background of research, Research Plan and Methodology:

Many references were cited but not listed. Many other references cited were not appropriate of showed in an inadequate critical awareness of what they did or did not support. For example, some citations were to a study on associations between exercise and mental health and did not support causal effects as the text indicated. Quite often the literature cited was dated or referred to a specific dated study when a current critical review of literature was needed. (eg, Gunnell, KE, et al (2019). Physical activity and brain structure, brain function, and cognition in children and youth: A systematic review of randomized controlled trials. MENPA, 16, 105-127; Lubans D, et al. Physical Activity for Cognitive and Mental Health in Youth: A Systematic Review of Mechanisms. Pediatrics. 2016 Sep;138(3). pii: e20161642.). Other references were misleading (eg, Costigan,et al, 2016) as it was claimed that exercise improved well-being when in fact there was no significant effect. Overall the rationale was not convincing with a lack of detailed discussion on why this population, why this intervention what the control group would receive and which outcomes would be most appropriate. The heavy reliance on Ranjbar et al. (2015) to guide the intervention was a worry since this was not one of the most rigorous reviews, but more a clinical opinion without critical appraisal.

I was concerned how the sample would be recruited without creating a social stigma for kids from low SES families. There was little mention of acceptability and feasibility issues from the underpinning pilot study. Which children would sign up for such a study? What would attendance and protocol adherence rates be like? What was described as work (eg, Doing push ups, squats and lunges at 85-95% for 15 secs 5 times for 4 circuits) is probably not likely to be sustainable for many, especially if they are inactive. Also, the protocol linked to a study by Wu was for adults.
In Table 1 there is reference to 'jumping jacks' being done for rest/recovery at 40-50% HR max. for 20-50 secs. Such exercise would normally be done at a much higher intensity than rest/recovery. I accept that the researchers have already done a pilot study but it would have been useful to hear if this has led to adaptations in the intervention protocol to demonstrate learning. A stronger evidence for possible effects of HITT for children would have been valuable. I was also unclear about the logistics of measuring HR and RPE for short bouts of exercise.

Measures: I suggest that rather than using a wide range of cognitive tests it would be better to be more focused on tests that have most relevance for the population of interest and articulate how they are linked to poor cognitive functioning in low SES (eg, inhibition and impulsivity may be linked to ADHD and substance use). I am not convinced that enjoyment is a good measure of well-being. It seems directed to exercise feelings which are simply subjective responses to exercise rather than any global measure of general well-being. What about the WEMWBS (see: Gireesh A1, Das S2, Viner RM1 Impact of health behaviours and deprivation on well-being in a national sample of English young people. BMJ Paediatr Open. 2018 Nov 9;2(1):e000335.). Self-efficacy: Seems like this is a measure of locus of control. I am not sure how this measure relates to the intervention. I would have thought that a measure of global self-esteem or physical self-perceptions may be more appropriate. No evidence was cited to suggest this global self-efficacy measure, or the Ill-being measure may be related to exercise in kids, or what the size of effects may be to calculate sample size. This mood scale was originally designed to capture acute changes in mood but has been discredited by Ekkekakis and colleagues for this purpose. Not clear why

the physiological measures are being taken. Will mediation analysis be conducted to determine if such outcomes mediate intervention effects.

4. The feasibility of the proposed research:

My guess is that it probably needs much longer to write a protocol and participant facing recruitment materials for ethical approval.

Without any specific mention of the timing of follow-up (other than 'post-intervention') I assume it will be immediately after the 10 week intervention. It would be more beneficial to know if any effects last longer than this, so adding a later follow-up assessment (eg, 3 or 6 months) would give an indication of persistence of effects, and limit the immediacy effects from having just completed the final exercise session.

The sample size calculations make no reference to clustering and this would typically lead to a need for a larger sample size than a simple 2-arm RCT.

5. The most original or innovative aspect of the proposed research; the research result bring about to the related field if the proposed research is successful:

A focus on those from low SES, with generally a higher prevalence of poor mental health.

6. The reasonableness of the proposed budget, manpower planning and project duration:

I think the research should include a longer follow-up period.

7. Overall Assessment
a) Overall Comments:

Generally a rather rushed proposal which needs more rigor in constructing a rationale, and choosing the methods and intervention. Assessing HITT interventions is somewhat of a fad at the moment and it is not clear what mechanisms might work in providing mental health benefits from HITT (v aerobic exercise v passive control).

b) Strengths:

There is a need to conduct more research involving children from disadvantaged groups.

c) Weaknesses:

The proposal overall lacked attention to detail in building a strong rationale, and adopting appropriate methodology.

d) Suggested improvements:

I think these are all covered in my comments above.

Proposal Assessment

The project:

Scientific/ scholarly merit:	Very Good
Duration proposed:	Appropriate
Impact of Research:	Moderate

The principal investigator:

Ability to undertake the proposal:	Very Good
Track record in field:	Very Good

Overall Recommendation: **Very Good**

DETAILED COMMENTS

1. The objective(s):

The project entitled Effects of Aerobic Exercise and High-Intensity Interval Training on the Mental Health of Adolescents Living in Poverty has a twofold purpose: a) To examine a 10-week exercise program targeting three mental health indicators (i.e. reduced ill-being/ enhanced well-being/ enhanced cognitive function) in low SES adolescents, and b) to compare the effects between aerobic exercise and high intensity interval training (HIIT) on these three mental health indicators in low SES adolescents.
While exercise has been linked positively to improved mental health, few studies have examined the population of adolescents and do not mention about the low SES adolescents. In addition, few studies to date have compared the different types of exercise (e.g., aerobic exercise and HIIT) on mental health in a special population, which might able to provide the necessary information regarding the specific exercise programs required to increase mental health in a special population. That is, the project provides an adequate address of those issues regarding the importance for conducting research from both academic and social impact perspectives.

2. Policy relevance of the proposal on public policy in Hong Kong:

One of the main issues in the project focuses on adolescents with low SES, a population that requires more attention. As stated in the project, adolescents' poor mental health has serious implications for adult morbidity and mortality, since nearly 49% of secondary students had suicidal thoughts, while an alarming 86% also had a negative self-image; nearly 75% of them experienced low self-worth in Hong Kong (HKJC, 2017). Notably, poor mental health is not uniformly distributed across socioeconomic groups, but is highly clustered in families with low socioeconomic status (SES) (Karlsen, Clench-Aas, Roy, & Raanaas, 2014), suggesting the importance for conducting research in Hong Kong.
The project is also timely because that population likewise has less physical activity habits. That is, 96% of the adolescents in Hong Kong are insufficiently physically active (HKSAR, 2018).
Accordingly, the project would meet the goal of informing public policy on health concerns in Hong Kong.

3. The Background of research, Research Plan and Methodology:

The Background of the research, the Research Plan and the Methodology of the project are adequate, while providing a sufficient rationale, based upon updated scientific evidence. On the other hand, the linkage regarding the importance of comparing two types of exercise regimens, requires a stronger rationale.

4. The feasibility of the proposed research:

The approach of the project is concerned with feasibility. At the same time, please note that the adolescents might be bored with a typical aerobic exercise regimen, used for conducting receptive and unconscious movements. Additionally, regarding HIIT, participants might experience negative emotions during the high intensity exercise, which might reduce their committed interests. That is, along with examining the effects of the exercise regimen, the project could additionally concern itself with building an exercise program that will attract the targeted population.

5. The most original or innovative aspect of the proposed research; the research result bring about to the related field if the proposed research is successful:

The most original or innovative aspect of the project is to focus on the special population of adolescents with low SES, as well as comparing two different exercise regimens, in order to develop the latter program so as to improve the mental health in the population.

6. The reasonableness of the proposed budget, manpower planning and project duration:

The proposed budget, manpower planning and project duration are reasonable.

7. Overall Assessment

a) Overall Comments:

The overall comments is that the project is in the Very Good shape. Other comments please see above and below.

b) Strengths:

The strengths of the project are to a) examine the special population (i.e., adolescent with low SES) that affect Hong Kong seriously; b) compare the effectiveness of the exercise program in order to improve the special populations' mental health; c) provide the scientific evidence necessary for selecting potential exercise programs for the special population.

c) Weaknesses:

The main innovation of the project is to examine the effectiveness of the exercise on mental health in a special population that is less frequently examined. It should be noted that the effects of exercise programs on specific mental health conditions have been demonstrated in a variety of researches, and therefore, the project might have less impact on knowledge from mechanism perspectives.

d) Suggested improvements:

Three suggestions are as follows:
a) provide a stronger rationale when comparing the two type of exercise regimens,
b) along with examining the effectiveness of the exercise program, also considering the exercise program from the perspective of how to attract the targeted population into participating in the study,
c) increase the KPI regarding publishing the project into scientific international journals, rather than only providing reports in newspapers and at press conferences.
